# Daily Evolution of Insulin Sensitivity Variability with Respect to Diagnosis in the Critically Ill

**DOI:** 10.1371/journal.pone.0057119

**Published:** 2013-02-21

**Authors:** Tamás Ferenci, Balázs Benyó, Levente Kovács, Liam Fisk, Geoffrey M. Shaw, J. Geoffrey Chase

**Affiliations:** 1 Department of Control Engineering and Information Technology, Faculty of Electrical Engineering and Informatics, Budapest University of Technology and Economics, Budapest, Hungary; 2 Department of Mechanical Engineering, Centre for Bio-Engineering, University of Canterbury, Christchurch, New Zealand; 3 Department of Intensive Care, Christchurch Hospital, Christchurch School of Medicine, University of Otago, Christchurch, New Zealand; Queen's University Belfast, United Kingdom

## Abstract

**Introduction:**

This study examines the likelihood and evolution of overall and hypoglycemia-inducing variability of insulin sensitivity in ICU patients based on diagnosis and day of stay.

**Materials and Methods:**

An analysis of model-based insulin sensitivity for 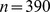 patients in a medical ICU (Christchurch, New Zealand). Two metrics are defined to measure the variability of a patient's insulin sensitivity relative to predictions of a stochastic model created from the same data for all patients over all days of stay. The first selectively captures large increases related to the risk of hypoglycemia. The second captures overall variability. Distributions of per-patient variability scores were evaluated over different ICU days of stay and for different diagnosis groups based on APACHE III: operative and non-operative cardiac, gastric, all other. Linear and generalized linear mixed effects models assess the statistical significance of differences between groups and over days.

**Results:**

Variability defined by the two metrics was not substantially different. Variability was highest on day 1, and decreased over time (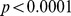) in every diagnosis group. There were significant differences between some diagnosis groups: non-operative gastric patients were the least variable, while cardiac (operative and non-operative) patients exhibited the highest variability.

**Conclusions:**

This study characterizes the variability and evolution of insulin sensitivity in critically ill patients, and may help inform the clinical management of metabolic dysfunction in critical care.

## Introduction

Stress induced hyperglycemia is a significant issue in critical care, affecting up to 30–50% of patients and increasing morbidity and mortality [1], [2]. Controlling glycemia has proved difficult due to the associated risk of hypoglycemia when highly dynamic patients are treated with exogenous insulin [3]. Both extremes, as well as glycemic variability, have been independently linked to increased morbidity and mortality [4]–[6], creating a difficult clinical problem.

More specifically, inter- and intra- patient metabolic variability drive outcome glycemic variability and hypoglycemic risk [7] making good control difficult. In particular, sudden and large rises in insulin sensitivity can result in a hypoglycemic event when exogenous insulin is given over a typical 3–4 hour measurement interval. It is critical to determine the size and likelihood of these intra-patient variations, to enable a more complete understanding of the inherent risks in glycemic control.

Very few studies have examined time-varying evolution of insulin sensitivity and its variability in the critically ill. Langouche et al noted [8] that insulin sensitivity rose between days 1 and 5 over their large cohorts, but provided no daily or diagnostic specific evolution. Lin et al showed [9] that hour to hour changes for a clinically validated model-based insulin sensitivity metric could be quite large as a function of current insulin sensitivity level for a medical Intensive Care Unit (ICU) cohort that covered all diagnostic categories and days of ICU stay. However, no studies to date have explicitly described the evolution of intra-patient insulin sensitivity and its variability on a daily basis, or for different diagnostic categories.

Such information would provide insight into the risk of hypoglycemia by diagnostic category and day of ICU stay. Additionally, insight into the likelihood of glycemic variability resulting from greater or lesser intra-patient variability of insulin sensitivity could be attained. This research presents a first rigorous statistical analysis of inter- and intra- patient insulin sensitivity variability as a function of diagnostic category and day of stay.

## Materials and Methods

### Ethics statement

The Upper South Regional Ethics Committee, New Zealand, granted ethics approval for the audit, analysis, and publication of these data. Data collection is described in detail in [10].

### Patient data

Clinical data from 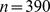 patients (47,836 hours) in the SPRINT medical ICU cohort [10] are used to identify hourly, model-based insulin sensitivity (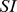) values (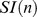). SPRINT is a model-based, clinically validated tight glycemic control (TGC) protocol that provides explicit control for both nutrition intake and insulin input [10].

Hour-to-hour changes are evaluated for the cohort over all days of ICU stay using a stochastic model [9] that provides kernel density estimation-based distributions of 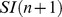 values for each current 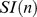 value using all 47,836 data points. Table 1 shows the patient demographic details, including diagnostic categories. These were created based on the APACHE III codes, and consist of operative and non-operative groups for cardiac, gastric and all other patients (with abbreviations OpC, NOpC, OpG, NOpG, OpO and NOpO, respectively). For the daily statistics, only patients who had at least 24 hours of glycemic control and ICU stay were used.

**Table 1:** Demographic data of patients.

Group	Day 1			Day 2		
	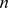	Age	Sex	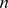	Age	Sex
NOpC	28	59.5 (61.5) 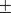 16.5 (24)	35.7	18	58.4 (59.5) 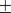 16.1 (19)	38.9
OpC	35	72.9 (73) 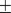 7.12 (10.8)	22.9	21	72.9 (73) 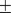 6.54 (10)	23.8
NOpG	16	64.3 (67) 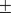 12.8 (15)	25	13	64.4 (71) 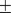 14.2 (18.5)	23.1
OpG	42	67.9 (72) 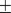 12.4 (13)	35.7	29	69.9 (72) 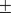 10.8 (11.3)	27.6
NOpO	119	54.7 (59) 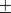 18 (27)	46.2	101	54.5 (59) 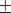 18 (28)	42.6
OpO	21	50.8 (56) 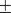 19.2 (31)	38.1	16	54.9 (57.5) 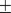 18.5 (31)	43.8

The distribution (according to length-of-stay and diagnosis group) and the most important demographic indicators of the patients. Data are shown in an 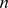, age, percentage of females format, with age statistics arranged in Mean (Median) 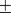 SD (IQR) manner. Columns indicate minimum (and not exact) length-of stay, so the same patient may appear in several cells.

### Variability metrics

Actual 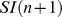 values for each day of ICU stay and each diagnostic category (cardiac, gastric, all other, both operative and non-operative in all three types) are compared to the distributions provided by the stochastic model of Lin et al [9] that covers all diagnostic categories and all days of ICU stay. The results thus show the relative and absolute evolution of 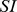 variability (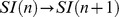) for a given diagnostic category over time, relative to all patients and days of stay, which should highlight times or diagnostic groups with greater or lesser than average risk.

The percentile of the actual 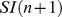 values on their predicted distribution will be illustrated with histograms. If the prediction is perfect (that is, the distribution of actual values is identical to the predicted distribution), every 10% wide interval of the histogram contains 10% of the measurements. This ideal case therefore corresponds to a flat distribution. Kurtic distributions are seen when the actual values were more concentrated at the median than the predicted distribution, suggesting confidence bands could have been tightened. In contrast, U-shaped distributions indicate cases where confidence bands should be widened due to increased variability.

Two metrics are used to assess variability for each patient over a given day, and results are aggregated by diagnostic category. First, a quadratic metric is defined as the average of squared deviations of the percentile of the actual 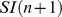 value on its predicted distribution (from the overall cohort model) from the ideal 50th percentile. This value increases the more variable a given patient. The quadratic metric thus measures overall intra-patient variability.

Second, a one-sided threshold metric counts the number of 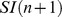 values for a given patient that exceed the 90th percentile of 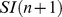 in the whole-cohort model of Lin et al [9]. This metric thus counts the number of large positive changes in 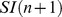 that would induce large drops in glucose level on dosing exogenous insulin based on the 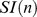 value. A value greater than 10% for a given patient, day or diagnostic category indicates a greater risk for these changes compared to the overall cohort on all days of ICU stay. This metric thus specifically assesses hypoglycemic risk due to intra-patient variability in insulin sensitivity and its daily evolution.

Hence, these two metrics measure overall variability and hypoglycemic risk from variability. Clinically, the quadratic measure is one of risk to glycemic control performance and outcome arising due to variability in insulin sensitivity, and the one-sided threshold assesses risk to patient safety in glycemic control.

**Figure 1:**
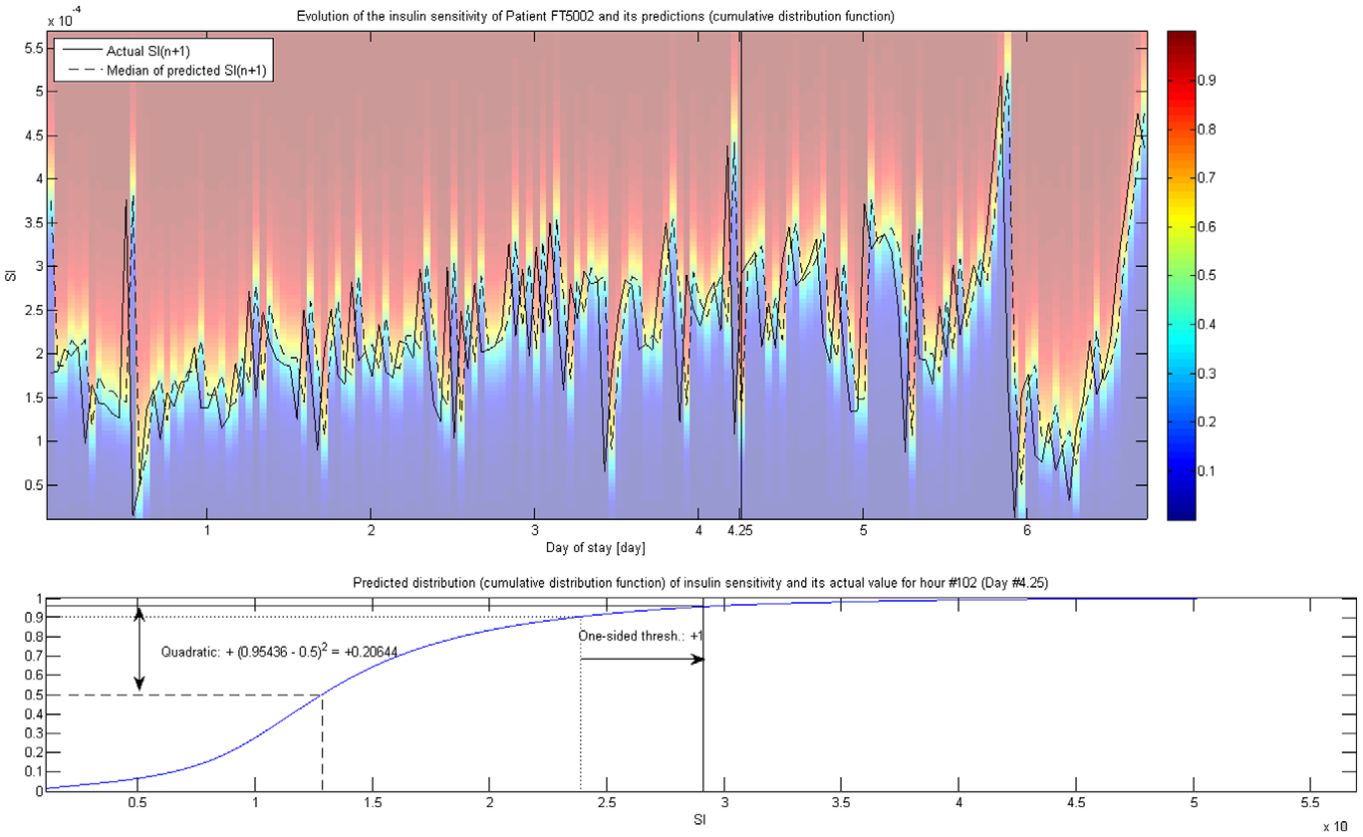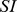 variability and its metrics. Illustration of the evolution of 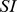 for a given patient (FT5002). Background colors represent the cumulative distribution function of the prediction for 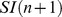 based on 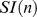 using the whole cohort; its 25th, 50th (i.e. median) and 75th percentile is explicitly shown. Lower part of the Figure highlights the calculation of the two metrics using Hour #102 (Day #4.25, marked on the upper part) as an example.

These metrics are illustrated on Figure 1, which shows the evolution of the insulin sensitivity of a 67 years old male patient (FT5002) with septic shock principal diagnosis (all other, non-operative category) through 162 hours. Each patient has such a trajectory. For every hour, the distribution of 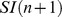 was predicted based on 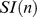 using the model of Lin et al [9], which is illustrated with the underlying colormap representing the cumulative distribution function of the predicted distribution. 50th percentile (i.e. median) of this predicted distribution of 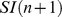 is explicitly shown. The Figure also illustrates how these metrics are calculated, showing the predicted distribution and the actual 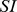 for a given hour.

### Analysis of variability

An overall variability score can be calculated for a given diagnosis group by averaging the overall variability scores for patients belonging to that group. However, if the individual length of stay differs, simple arithmetic averaging would assign unequal weights for each patient's measurements. To avoid the problems associated with unequal weighting due to patient discharge, only series of equal length were averaged. In particular, results and analysis were divided by the first 24 hours (“day 1”), second 24 hours (“day 2”), third 24 hours (“day 3”), and remaining time in ICU (“day 4+”). Thus only complete 24 hour intervals were used (except for day 4+, of course) to avoid bias.

Per-patient average penalty score distributions by diagnosis group each day are shown using violin plots [11]. Violin plots bear similarities to boxplots, but use kernel density estimation to directly convey information on the shape of the distribution for more accurate comparison.

### Statistical methods

**Figure 2:**
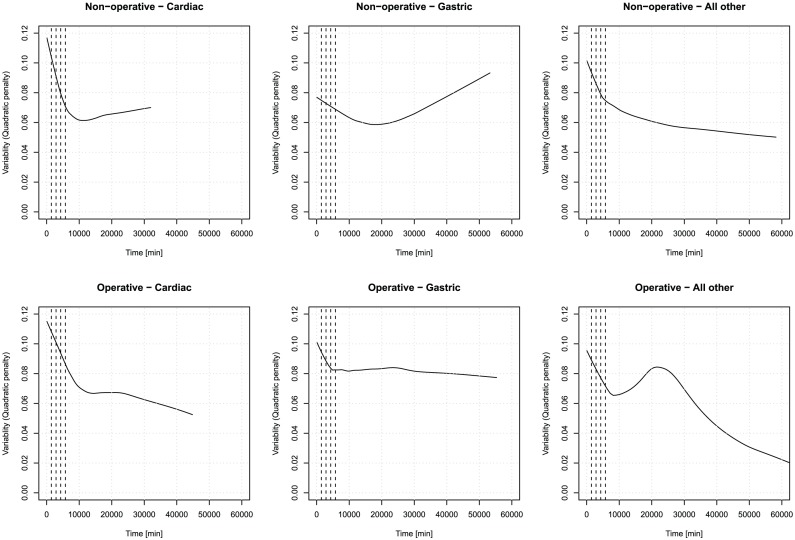
Evolution of quadratic 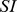 variability per diagnosis group. LOWESS estimators for the scatterplot between minute-precision length of stay and quadratic metric of 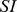 variability, segregated according to diagnosis group. Dashed vertical lines indicate the end of the first four days.

To have an overall impression on the effect of the time spent in ICU on the 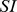 variability, a LOWESS estimator [12] was plotted for the scatterplot of quadratic metric and time spent (in minutes) per diagnosis group on Figure 2. It is immediately obvious that time has a complex effect on 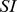 variability, which exhibits a biphasic behaviour in most of the cases. This might be worthy of pursuit, despite the fact that the estimation at long length of stays is unreliable due to relatively lower sample size.

However, now we will confine our attention to investigate the early, seemingly mostly linear response of the first few days. (To illustrate this, the first four day is marked on Figure 2.) We restricted the database to observations having Time 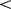 8 000 minutes (i.e. the first 5.5 days of stay) for the estimation of the forthcoming models, hence limiting it to the “linearity region” of the 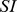 variability vs. time function, as evidenced by Figure 2. The linear functional form is also more tractable and easier to estimate.

To account for the grouping of the data, linear mixed-effects modelling was used to find significant differences in 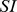 variability metrics between diagnosis groups and/or days [13], [14]. The (longitudinal) data were arranged in a two-way classification, with time a within-subject factor and diagnosis group considered a between-subject factor. In our model, the fixed effects were the Time (time spent in ICU in minutes as a continuous variable) and the Diagnosis (as a nominal factor with 6 levels) without intercept (“cell means coding”). Minute-precision length-of-stay (Time) was used for measuring time to make the estimation of the mixed-effects model possible. The random effect was added with per-patient grouping, with both random intercept and random slope permitted with respect to time, both of which was deemed necessary with LR-test (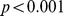 for both quadratic and one-sided penalty) [15]. The inclusion of an AR(1) autocorrelation of the within-subject errors was not found to be necessary for the quadratic penalty () [15]. The fixed effects interaction terms between Time and Diagnosis were found to be insignificant (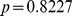 for quadratic penalty, 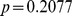 for one-sided penalty) showing that that the slope with respesct to the time spent in ICU does not depend on the diagnosis group, and were thus eliminated. (Effect of Diagnosis was significant (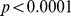 for both penalty), so the intercept does depend on the diagnosis group.) The resulting statistical model for the quadratic penalty of 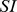 variability was therefore the following:
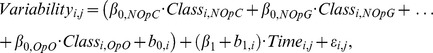
(1)where 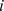 identifies the patient, 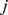 identifies the measurement (i.e. 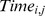 is the time of the 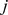
th measurement on patient 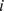), 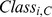 is the indicator variable for Class 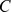 (i.e. takes the value of 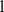 if patient 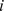 is in class 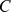, 0 otherwise). For the one-sided threshold penalty – as the response is essentially binary – generalized linear mixed effects (GLME) modeling [16] was used instead of the traditional linear mixed effects (LME) modeling. The link function was chosen to be logistic, and the distribution family was binomial. For the quadratic penalty, LME modeling was used, but the penalty score was (monotonically) logit-transformed beforehand to map the skewed distribution on 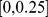 to an approximately normal one on the real line [15]. This sacrifies the interpretability of the coefficients for the correct specification of the model, but the former was of little concern for us, as we will not use the numerical values of the coefficients for further analysis. Linearity for the transformed data was still feasible.

The coefficients are denoted with 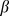 for the fixed, and with 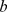 for the random effects. The fixed effects coefficient of Time characterizes – for the whole population – how variability changes over time, with positive value implying increasing variability, negative implying decreasing variability, and the absolute value showing the size of this effect. The fixed effects coefficients of diagnosis groups show the estimated variability of a patient in the given diagnosis group when admitted to the ICU.

Restricted maximum likelihood (REML) was used for the estimation of LME models and Laplace-approximation for GLME. Residual variance was rather high in both cases, indicating that the models were only able to capture a small part of the variation – but this is to be expected, given that we use no information other than time spent in ICU and diagnosis group.

After performing ANOVA to assess the significance of main effects, post-hoc testing on significant effects was carried out using Tukey's Honestly Significant Differences (HSD) method [17], providing the correction that takes the multiple comparisons situation into account.

### Data processing

Data processing was done using Mathworks Matlab (version 2009a). Statistical analysis was performed under the R statistical program package [18], version 2.15.1 with nlme package for LME modeling [19] and lme4 package for GLME modeling [20].

## Results

**Figure 3:**
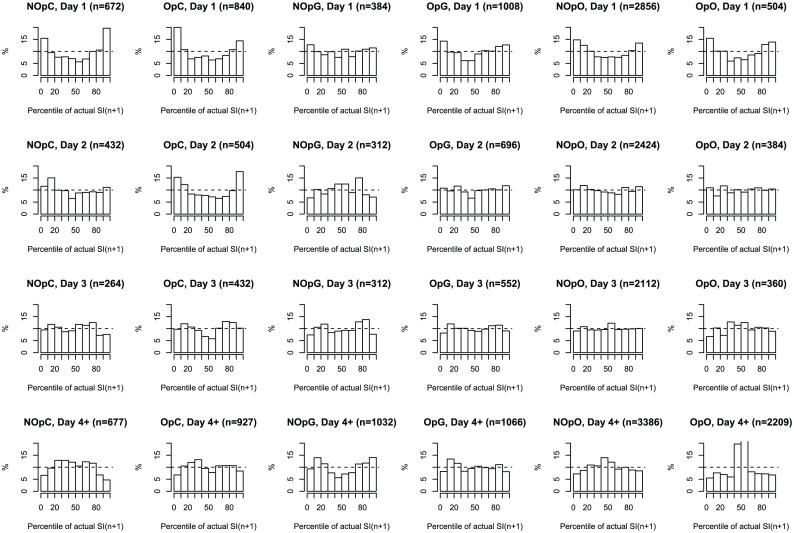
Distribution of predictions according to diagnosis and day of stay. Histograms of the percentile of actual 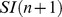 values on their predicted distribution grouped according to day (rows) and diagnosis group (columns). Dashed line indicates the ideal (uniform) case of perfect prediction. The number of hourly measurements which was used to construct the histogram is shown in the title.

**Figure 4:**
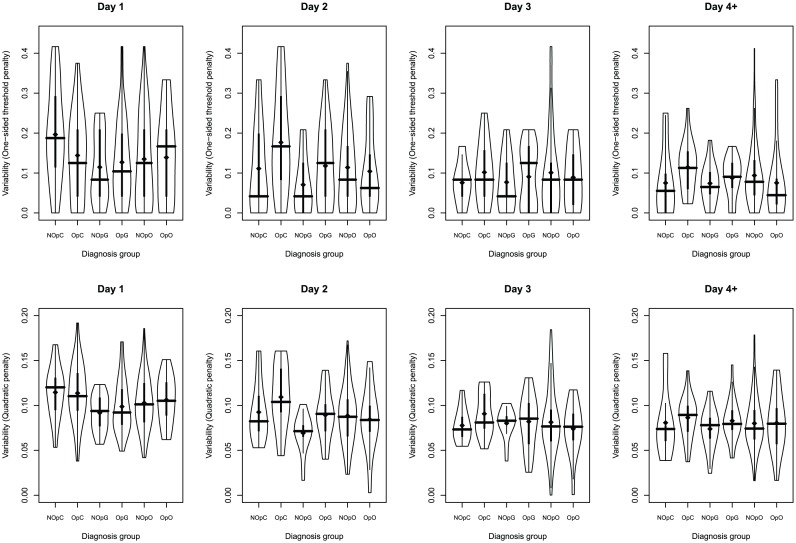
Distribution of per-patient variability scores according to diagnosis and day of stay. Violin plots of per-patient overall variability scores segregated according to day and diagnosis group. Upper row shows one-sided threshold penalty metric, while lower row shows the quadratic penalty metric. Thick vertical lines indicate the interquartile range, the crossing horizontal line is at the median. Dots indicate the mean.

Figure 3 shows the distribution of the percentile of actual 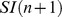 on its predicted distribution for different days and diagnosis groups. Figure 4 shows the violin plot of the distributions of per-patient overall variability metrics in different diagnosis groups, segregated according to ICU day and diagnosis group. Parameters of the fitted GLME model (for one-sided threshold penalty) and LME model (for quadratic penalty) are shown in Table 2.

**Table 2:** Fixed effect coefficients of the fitted models for the one-sided and the quadratic penalty.

Variable	One-sided penalty	(Transformed) Quadratic penalty
Non-operative - Cardiac	−1.5807	−0.5033
Operative - Cardiac	−1.9092	−0.4427
Non-operative - Gastric	−2.3532	−1.048
Operative - Gastric	−1.8791	−0.6922
Non-operative - All other	−1.9903	−0.7350
Operative - All other	−2.0911	−0.8467
Time (per minute)	−0.00008571	−0.0001257
Time (per day)	−0.1234224	−0.1810
	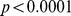	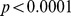

Summary of the estimated fixed effect coefficients of the LME model for (logit-transformed) quadratic penalty and the GLME model for the one-sided threshold penalty, and the 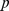-value for the test of significance for Time. The coefficient of Time is given both per minute and per day (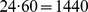 times the former).

The distributions in Figure 3 suggest poor coverage of the whole-cohort model on day 1, almost ubiquitously across diagnosis groups. On day 2, every diagnosis group “flattens”, except for Operative - Cardiac. On day 3, the predictions are acceptable in every diagnosis group in that the actual distribution of 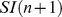 largely matches the whole cohort-predicted distribution. Finally, on day 4 and onwards the coverage is very over-conservative in the Operative - All other category.

Figure 4 (top row) suggests that one-sided threshold penalties exhibit much larger, typically positively-skewed variations. There is a slight trend in the central tendency, as median variability in this metric appears to decrease as time increases. A trend towards reduced spread in this (one-sided) variability over time is more pronounced, indicating decreasing risk of hypoglycemia over time when all else is equal.

In contrast, quadratic penalties are much more centrally concentrated, and have a smaller coefficient of variation. The continuous lowering of variability over time in every group is also seen, but a reduction in spread is not as pronounced. The two metrics are consistent in assigning “higher” and “lower” variabilities similarly over time and diagnostic group, albeit on different scales.

As can be seen from Table 2, time trend was significant (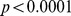) with a coefficient of 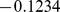/day for the one-sided threshold penalty, and 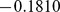/day for the (transformed) quadratic penalty, indicating the decreasing variability over time in both cases. These results also imply a decreasing risk of hypoglycemia inducing variability in insulin sensitivity over time, matching trends in Figure 4.

Post-hoc testing for diagnosis groups also revealed significant differences. Using Tukey's HSD method (see Table 3), Non-operative - Cardiac group had significantly (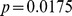) higher variability than Non-operative - Gastric for the one-sided threshold penalty. Non-operative - All other category also exhibited marginally significantly (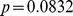) lower 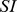 variability than Non-operative - Cardiac patients. The Operative - Cardiac exhibited significantly (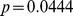) higher variability than Non-operative Gastric for the (transformed) quadratic penalty. These results suggest that the Non-operative - Gastric group is amongst the least variable groups, while the Cardiac groups exhibit the highest variability irrespective of day. These results are consistent with Figure 4, though it is worth noting that cardiac patients “change place” from day 1 to day 2 irrespective of penalty: Non-operative - Cardiac patients are more variable than Operative - Cardiac group on day 1, but this order is reversed from day 2 onwards.

**Table 3:** Significance of the effect of diagnosis group with Tukey-HSD correction for multiple comparisons.

Comparison	One-sided penalty		(Transformed) Quadratic penalty	
	Estimate	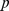	Estimate	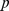
OpC - NOpC	−0.3285	0.4188	0.0606	0.9992
NOpG - NOpC	−0.7724	0.0172	−0.5451	0.1505
OpG - NOpC	−0.2984	0.5130	−0.1889	0.8637
NOpO - NOpC	−0.4096	0.0835	−0.2317	0.6190
OpO - NOpC	−0.5104	0.1438	−0.3434	0.5038
NOpG - OpC	−0.4440	0.3607	−0.6057	0.0444
OpG - OpC	0.0300	1.0000	−0.2495	0.4946
NOpO - OpC	−0.0811	0.9890	−0.2923	0.1525
OpO - OpC	−0.1819	0.9335	−0.4040	0.2077
OpG - NOpG	0.4740	0.2765	0.3563	0.5179
NOpO - NOpG	0.3628	0.5024	0.3135	0.5799
OpO - NOpG	0.2621	0.9034	0.2017	0.9539
NOpO - OpG	−0.1112	0.9503	−0.0428	0.9992
OpO - OpG	−0.2120	0.8732	−0.1545	0.9518
OpO - NOpO	−0.1008	0.9919	−0.1117	0.9817

Estimates of differences and the 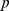-values for the test of their significance (using Tukey-HSD post hoc testing for the multiple comparisons situation) for the pairwise comparison of diagnostic categories.

## Discussion

Clinically, those results indicate a decreasing likelihood of hypoglycemia induced by large rises (variations) in insulin sensitivity over short measurement and intervention intervals as days of ICU stay increase based on the one-sided threshold results. The overall risk of increased variability of both forms (one-sided and quadratic metrics) by diagnostic category is highest for Cardiac patient groups.

This latter observation is matching the increased hypoglycemia observed in glycemic control studies in these cohorts (e.g. [21]). The highest variability on day 1 is consistent with the increased hypoglycemia and range observed in the first 24 hours in the study by Bagshaw et al [4], which was associated with increased risk of death. The overall higher variability (quadratic measure) on day 1 in all groups is also reflective of increased hypoglycemia and variability reported in most glycemic control studies irrespective of cohort [3], [4].

The major strength of the present study is that it also provides a rigorous statistical framework, which makes the quantification of these effects possible. It is, however, limited in some sense because it is inherently linked to the SPRINT protocol (as it interprets variability as the deviation of the actual 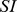 from its prediction provided by the particular algorithm in that protocol).

The physiological causes of this variability have links to the counter-regulatory and oxidative stress responses, and inflammatory acute immune response typically seen in hyperglycemic critically ill patients. That the variability declines over days 1–4 as the acute phase passes also matches expectations and physiological observations. Drug therapies, such as glucocorticoid or inotrope use [22] among others, may also be implicated as a causative factor. However, the high level of patient-specificity observed within any group makes determining specific causes or magnitude of effect difficult.

For glycemic control, high levels of variability combined with infrequent BG measurement are a major disincentive to higher insulin doses and/or low glycemic targets. The only study to reduce both mortality and hypoglycemia [10] was notable in modulating both insulin and nutrition inputs to achieve good control with lesser insulin and thus reduce hypoglycemic risk. Hence, either higher targets [23] and/or adding nutritional intake into consideration in providing glycemic control [24] must be considered for at least some diagnostic groups (e.g Cardiac patients) and days of ICU stay (day 1) based on these results.

## Conclusions

Inter-patient variability in insulin sensitivity peaks on day 1 across diagnostic groups and metrics. Operative - All other patients are more predictable after day 4 than an all patients and days of stay model accounted for, shown by conservative coverage. The distribution of overall intra-patient variability assessed per-patient and the mixed-effects model shows there are distinctive differences between diagnosis groups, irrespective of the time spent in the ICU. In particular, the Non-operative - Gastric group exhibits the smallest variability, while Cardiac groups are amongst the most variable. Clinically, these results show decreasing risk of hypoglycemia as length of stay increases, as well as some reduction in glycemic variability when all else is equal. The overall results can be used to guide the design and implementation of glycemic management specific to diagnosis group and ICU day of stay to improve control and reduce risk.
